# Dimensionality engineering of hybrid halide perovskite light absorbers

**DOI:** 10.1038/s41467-018-07382-9

**Published:** 2018-11-28

**Authors:** Peng Gao, Abd Rashid Bin Mohd Yusoff, Mohammad Khaja Nazeeruddin

**Affiliations:** 1 CAS Key Laboratory of Design a Assembly of Functional Nanostructures, and Fujian Provincial Key Laboratory of Nanomaterials, Fujian Institute of Research on the Structure of Matter, Chinese Academy of Sciences, 350002 Fuzhou, Fujian China; 2 Group for Molecular Engineering of Functional Materials, Institute of Chemical Sciences and Engineering, École Polytechnique Fedérale de Lausanne, 1951 Sion, Switzerland; 3 Advanced Display Research Center, Department of Information Display, Kyung Hee University, Dongdaemoon-gu, 130-701 Seoul Korea

## Abstract

Hybrid halide perovskite solar cells were first demonstrated in 2009 with cell efficiency quickly soaring from below 10% to more than 23% in a few years. Halide perovskites have the desirable processing simplicity but are very fragile when exposed to water and heat. This fragility represents a great challenge for the achievement of their full practical potential in photovoltaic technologies. To address this problem, here we review the recent development of the mixed-dimensional perovskites, whereby the trade-off between power conversion efficiency and stability of the material can be finely tuned using organic amine cations with different sizes and functionalities.

## Abstract (web summary)

Organic−inorganic metal halide perovskite solar cells possess high efficiency and low processing cost but suffer poor stability. Here Gao et al. review the recent progress on the 2D–3D mixed perovskites and suggest that greatly improved stability can be achieved without compromising the efficiency.

## Introduction

**Fig. 1:**
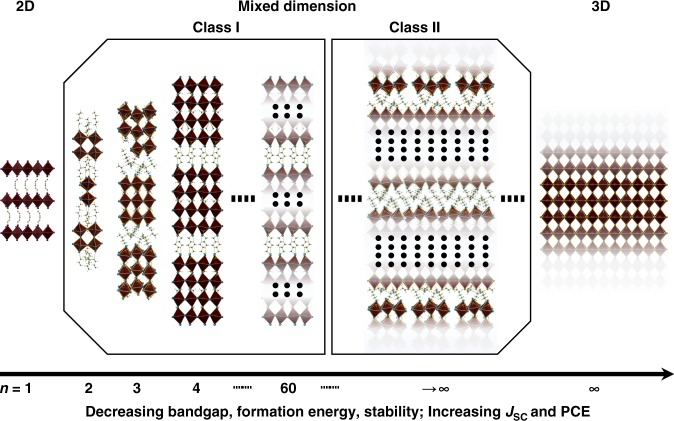
Dimensionality engineering. Effects of perovskite phase evolution on chemical and physical properties

The emerging perovskite solar cells (PSCs)^1^ have delivered power conversion efficiency (PCE) on par with that of polycrystalline silicon solar cells^2^, owing to the considerable research effort directed into the design and optimization of perovskite materials^3^. The efficiency race of PSCs has been accompanied by the questions concerning the real potential of PSCs for widespread deployment. When evaluating a PV technology with LCOE (Levelized cost of electricity), PCE, cost and lifetime are the three utmost criteria^4,5^. Since they determine the possibility of the technology to be accepted by the market. First and foremost, there come the concerns about the stability of intrinsic 3D hybrid perovskite materials. To ameliorate the notorious pitfall on the way, two main measures have been taken: providing sufficient protection to the perovskite material and increasing the intrinsic stability of the perovskite material. In the first case, a rich variety of methods have been developed: (1) exploration of moisture-resistant charge transporting materials and electrodes; (2) interface modification of the devices by interfacial resistant agents; and (3) proper encapsulation of the devices etc^6,7^. In the second case, three main strategies are typically used: (1) compositional engineering of halides and cations (mixing cations and/or anions, adding additives) within the 3D criteria of tolerance factor^8–10^; (2) formation of low-dimensional polymorphs on 3D perovskite as a protective layer; (3) formation of Ruddlesden–Popper type perovskite. The last two strategies to stabilize perovskite can be generally called multi-dimensional perovskite (MDP). In most instances, the gain in the long-term stability was accompanied by the sacrifice in the power conversion efficiency (PCE) except the strategies represented by mixing three-dimensional (3D) perovskites with low-dimensional (2D, 1D, or 0D) perovskites (LDP)^11–13^ to form MDP with a general formula of (A)_2_(CH_3_NH_3_)_*n*−1_M_*n*_X_3*n*+1_ (1 ≤ *n* ≤ ∞). A systematic evolution of the dimensionality of the perovskite structure can be seen from Fig. 1. Usually, inside a typical MDP film, perovskite phases with different n values and structural gradients from pure 2D perovskite to pure 3D perovskite coexist.

As one of the three main motivations^14^ behind the development of derivatives of hybrid halide perovskites, the MDP strategy showed the potential to provide parallelly improved stability and PCEs. The question: ‘Is the MD perovskite a compromise or breakthrough?’ accompanied the searching for an optimized condition for the best MDP composition^11,15^. Because except the stability, the bandgap and binding energy are also increasing with the decreasing of the dimensionality, while the conductivity and the figure of merits are decreasing (Fig. 1). In this review, we discuss how the hybrid perovskite material can be engineered to enable the discovery and design of more stable perovskite materials with less or no trade-off in PCE, focusing on tuning and mixing components with different dimensionalities (MDP approach) within the photovoltaically active region. We begin with an incomplete but representative summary of the photovoltaic performance of 1D, 2D, MD, and 3D perovskites as shown in Table 1, based on which we provide additional insights into how the two types (Class I and Class II) of MDP could be designed or deposited in different cases to achieve balanced performance. Comparing with other approaches to stabilize 3D perovskite materials (vide supra), the MDP approach has the advantages of convenient, less cost, and taking effect in situ from inside of the materials. However, the deposition condition of Class I MDPs with low *n* numbers (*n* equals 2 to 5) has to be judiciously controlled to have vertically aligned octahedron planes as the beneficial channel for charge transport (vide infra). Although most of the published works showed an apparently fixed *n* value, the film is actually formed of a mixture of perovskite domain with varied numbers, which can explain the seemingly contradictive *E*_g_s in Table 1. On the other hand, Class II MDPs films have a 3D perovskite body under the cover of LDP or non-perovskite polymorphs. They keep the low *E*_g_ inside while taking the advantage of high formation energy of the external LDP. The phase disorder inside the film is much less since the LD perovskites are supposed to distribute mainly over the surface. This model is however hard to be verified directly without sophisticated equipment like Grazing Incidence Wide Angle X-Ray Diffraction (GIWAXD). The effect of protection can be weakened if the coverage of the LD perovskite is not sufficient. To date, the MDP strategy provided the most touching stability test result under in operando conditions, albeit not the world record PCE. As we have discussed before, the use of MDP will lead to the widening of *E*_g_ and the increase of resistance if the LDP is not properly deposited. Also, the realization of vertically aligned MDP needs expertized conduction of the deposition procedure. These drawbacks compromised photocurrent in MDP based devices. At the end of the discussion, we offer not only retrospective insights of known results, but also the strategies to nudge the compromise further into a breakthrough.

**Table 1:** Photovoltaic performance and stability of 2D-MD-3D perovskites

Dimensions	Absorber	PCE (%)	PCE (*s*) (%)^a^	*E*_g_ (eV)	Stability test (*h*)^b^	Ref.
1D						
EA/PA	EAPbI_3_	0.26	—	2.2		^88^
	PAPbI_3_	0.016	—	2.4		
2D						
BA/BdA/PEI/PEA	BA_2_PbI_4_	0.124	—	2.3		^89^
	[NH_3_(CH_2_)_4_NH_3_]PbI_4_	1.08	—	2.37	*T*_74_ = 48	^90^
	(PEA)_2_PbBr_4_	—	—	3.0		^68^
	(PEI)_2_PbI_4_	—	—	2.3		^91^
MD						
Class I						
BA/MA	(BA)_2_CsPb_2_I_7_	4.84	—	2.2	*T*_92_ = 720	^89^
	(BA)_2_(MA)_*2*_Pb_3_I_10_	4.02	—	1.89		^12^
	(BA)_2_(MA)_*2*_Pb_*3*_I_10_	11.44	—	—	—	^61^
	(BA)_2_(MA)_3_Pb_4_I_13_	8.79	6.8	1.65		^66^
	(BA)_2_(MA)_3_Pb_4_I_13_	12.51	—	1.66	*T*_70_ = 2050 *T*_100_ = 2250 (Sealed)	^61^
	(iso-BA)_2_(MA)_3_Pb_4_I_13_	10.63	6.43	1.74		^63^
	(BA)_2_Cs_3x_(MA)_3-3x_Pb_4_I_13_^c^	13.68	—	1.62	*T*_89_ = 1400	^62^
	(BA)_2_(MA)_4_Pb_5_I_16_	8.05	—	1.6		^92^
	(BA)_2_(MA)_4_Pb_5_I_16_	8.71	—	1.83		^38^
HA/MA/FA/Cs	HA_2_MAPb_2_I_7_	0.34	—			^64^
	HA_2_FAPb_2_I_7_	1.26	—			^64^
	HA_2_CsPb_2_I_7_	0.10	—			^64^
PEI/MA	(PEI)_2_(MA)_6_Pb_7_I_22_	10.08	—	1.62	*T*_90_ = 500	^35^
PEA/MA^d^	(PEA)_2_(MA)_2_Pb_3_I_10_	4.73	—	2.1		^11^
	(PEA)_2_(MA)_2_Pb_3_I_10_	3.72	—	1.94	*T*_70_ = 1440	^93^
	(PEA)_2_(MA)_9_Pb_10_Br_29_	—	—	2.3	—	^68^
	(PEA)_2_(MA)_49_Pb_50_Br_151_	8.5	—	2.2	—	^68^
	(PEA)_2_(MA)_59_Pb_60_I_181_	15.4	—	—	*T*_74_ = 1440 (N_2_); *T*_85_ = 336 (55%)	^34^
ALA^e^/Cs/FA	ALA_2_(MA_0.14_FA_0.81_Cs_0.05_)_9_Pb_10_(I_0.85_Br_0.15_)_29_	16.5	—	—	*T*_95_ = 680 (30–40%, dark)	^49^
Class II						
PEI/MA	(MAPbI_3_)_1−*x*_[(PEI)_2_PbI_4_]_*x*_(*x* = 0.02)	15.2	—		*T*_84_ = 336	^94^
BA/FA/Cs^f^	(BA)_0.09_(FA_0.83_Cs_0.17_)_0.91_Pb(I_0.6_Br_0.4_)_3_	17.2	17.3	1.72	*T*_80_ = 3,880 (sealed) *T*_80_ = 1005	^53^
	(BA)_0.05_(FA_0.83_Cs_0.17_)_0.95_Pb(I_0.8_Br_0.2_)_3_	20.6	19.5	1.61	—	^53^
AVA(3%)/MA^f^	(AVA)_2_PbI_4_/MAPbI_3_	14.6	—	1.69	*T*_60_ = 300	^58^
	(AVA)_2_PbI_4_/MAPbI_3_ module	10.10	—		*T*_100_ > 10,000 (sealed)	^58^
IC_2_H_4_NH_3_/MA/FA ion exchange^f^	(IC_2_H_4_NH_3_)_2_(MA)_x(*n*−1)_(FA)_*y*(*n*−1)_Pb_*n*_I_3*n*+1_^g^	9.03	—	1.63		^59^
Interface/morphology (LPK)	(PEA)_1−*x*_(FA)_*x*_PbI_3_ (M_FA_/M_PEA_= 40)^f^	17.71	17.3	1.522	*T*_90_ = 384	^56^
	(PEI)_2_PbI_4_ /MAPbI_3_	15.37	—		*T*_90_ = 200	^91^
	MAPbI_3_/(PEA)_2_Pb_2_I_4_ 3D–2D graded	19.89	19.85	1.53	*T*_60_ = 720	^42^
	(PEA_2_PbI_4_)_0.017_(MAPbI_3_)	19.8	17.8	—	*T*_92_ = 1008	^95^
	MAPbI_3_/BA_2_MA_2_Pb_3_I_10_	11.49	—			^54^
	MAPbI_3_/(PEA)_2_(MA)_4_(Pb_5_I_16_)	16.84	—		*T*_92_ = 456	^54^
2D passivation^f^	FEAPbI_3_/MAPbI_3_	17.9	—		*T*_92_ = 2880	^40^
	BzA-FAPbI_3_ (benzylammonium)	19.2	19.0	1.48	*T*_50_ = 3000	^51^
	BA-treated MAPbI_3_	19.56	19.29	1.54	*T*_96_ = 100 (95 °C, N_2_, dark)	^41^
	(MA)_1−2*x*_(EDA)_*x*_PbI_3_ (*x* = 0.008)	18.6	18.1	1.59	*T*_75_ = 72	^55^
	NH_3_I(CH_2_)_8_NH_3_I (C8)	17.60	—	—	—	^57^
3D						
Mono-ions						
FAPbl_3_		13.24	—	1.48	*T*_0_ = 200 (light) *T*_90_= 500 (85 °C, dark)	^96, 97^
MAPbI_3_		16.1	—	1.56	*T*_95_=1440 (25 °C, 30–50% RH)	^98^
CsPbI_3_		1.8	5.6 10.5	1.73	*T*_85_ = 840 (dark, dry box)	^89, 99, 100^
MAPbBr_3_		8.1	—	2.2	*T*_93_ = 1000 (ambient condition)	^68, 101^
Mixed-ions						
FA_1−*x*_MA_*x*_PbI_3_		20.65	—	—	*T*_85_= 500 (light) *T*_90_= 500 (85 °C, dark)	^97^
CsFAMAPbI_3−*x*_Br_*x*_		20.4	20.5	—	*T*_95_= 1000 (60 °C)	^10^
RbCsFAMAPbI_3_		21.8	21.6	—	*T*_95_= 500 (85 °C, 1 Sun)	^102^
CsPbI_2_Br		7.7	6.7	—	*T*_87_= 250 (85 °C and 85% RH)	^87^
CsPb_0.9_Sn_0.1_IBr_2_		11.33	—	1.79	*T*_100_ = 1750 (sealed, RT) *T*_100_ = 336 (sealed, 100 °C) *T*_85_ = 50 (unsealed RT, 50–60% RH)	^103^

^a^Stabilized power output under non-operating conditions

^b^The time span to 80% of the post burn-in decay (T80), obtained under different lifetime test conditions

^c^*x* = 0.05

^d^Quasi-3D with large *n* value

^e^Allylammonium

^f^No observable new/alien phase

^g^4 min. dipping time

## The evolution of dimensionality in hybrid halide perovskite

**Fig. 2:**
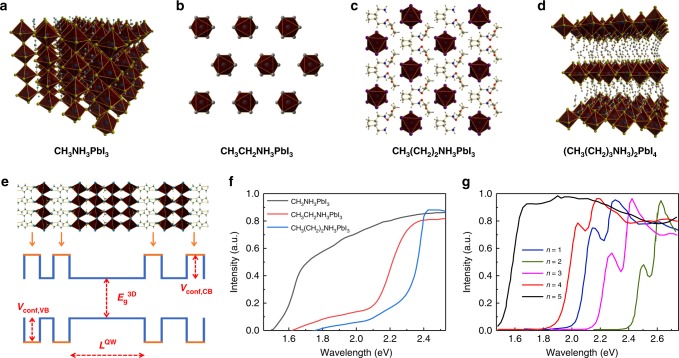
Cation dependence of the crystal structure of perovskite and the quantum confinement effect. **a** 3D networks of CH_3_NH_3_PbI_3_; **b** 1D chains of CH_3_CH_2_NH_3_PbI_3_; **c** 1D chain-structure of CH_3_(CH_2_)_2_NH_3_PbI_3_; **d** 2D layer-structure of CH_3_(CH_2_)_3_NH_3_PbI_3_. **e** Schematic MDP structure and possible energy-level schemes that can arise within these structures, where semiconducting inorganic sheets alternate with organic layers having much wider bandgaps, resulting in a Type I quantum well structure. UV−visible absorption spectra or Tauc plot of **f** MAPbI_3_, EAPbI_3_, and PAPbI_3_ (Reproduced with permission from ref. ^88^); **g** (BA)_2_(MA)_*n*−1_Pb_*n*_I_3*n*+1_ perovskites (for *n* =1, 2, 3, 4, ∞) (Reproduced with permission from ref. ^13^)

The most popular lead iodide based perovskite structure (rightmost panel of Fig. 1, Fig. 2a) consists of an extended three-dimensional (3D) network of corner-sharing PbI_6_ octahedra^16^. The organic cations fill the 12-fold coordinated voids among the octahedra. For a stable 3D perovskites, the suitable organic cation interact dynamically with the inorganic network to the extent that is imposed by the tolerance factor *t* defined by (*R*_A_ + *R*_X_) = *t*$\sqrt 2$ (*R*_M_ + *R*_X_), where *R*_A_, *R*_M_, and *R*_X_ denote the ionic radii of the three ions in AMX_3_^17^. Empirically speaking, perovskites with cubic crystal system should meet the requirement of 0.8 ≤ *t* ≤ 0.9, which allows a slightly extended range for distorted cases^18^. LDP features the structure of specifically cut or sliced 3D perovskite structure by specific organic cations (Fig. 2b–d). LDPs including layered perovskites and chained perovskites were of scientific interest owing to their prominent excitonic, ferroelectric, ferroelastic, and third-order nonlinear optical properties^19–22^. The polymorphs of chained perovskites can be further classified into face-sharing chains of PbI_6_ octahedra and corner-sharing chains of PbI_6_ octahedra^23,24^. Based on the 3D structure in Fig. 2a, if one more –CH_2_ is added to the organic cation, the 3D structure is destroyed and 1D chain-structure forms. Adding another –CH_2_ will not change the situation (Fig. 2b, c). Until *n*-butyl-ammonium is used as the cation, 2D layered structures formed, corresponding to the case of *n* equals 1 and A site cation of butyl group in (A_2_M_*n*_X_3*n*+1_) (*n* is an integer, A is an aliphatic or aromatic primary ammonium, M is a divalent metal, and X is a halide anion) (Fig. 2d). When we combine the size constrained cations with the sterically hindered cations, MDPs formed. Stoumpos et al.butyl-ammonium^13^, managed to solve the crystal structures of (BA)_2_(MA_)*n*−1_Pb_*n*_I_3*n*+1_ (BA: butyl-ammonium; MA: methyl ammonium; *n* equals 1 to 5). The visualized structure is beneficial to understand the stage of quasi-2D layered perovskite during the transition from monolayer 2D perovskite to infinite 3D perovskite after tuning the organic cation and stoichiometry of precursors. Moreover, almost all the MDP compounds reported to date are somewhere in between these cases.

It has been proved that the dimensionality can be harnessed to influence optical bandgap (*E*_g_) of the perovskite material^25^. Especially, the *E*_g_ values of MDPs are directly influenced by the 2D confinement effect^26–28^, of which the organic–inorganic Quantum Well structure of an MDP is shown in Fig. 2e. For a Ruddlesden–Popper hybrid perovskite, the *E*_g_ is dependent on the well-width (*L*^QW^)^29^ and the total *E*_g_ energy is determined by the *E*_*g*_3D and extra quantization energies of the electron and hole^30^. For MAPbI_3_ in all the reported works, a band edge of 785 nm and a bandgap of 1.6 eV were measured, which is in agreement with each other and previous studies^31^. For EAPbI_3_ (EA: ethyl ammonium) and PAPbI_3_ (PA: propyl ammonium) with 1D chained structure, this value has been shifted to 568 and 523 nm, corresponding to a bandgap of 2.2 and 2.4 eV, respectively (Fig. 2f). Moreover, there exists a significant dependence of *E*_g_ in the MDP (quasi-2D layered) perovskite ((A)_2_(CH_3_NH_3_)_*n*−1_MX_3*n*+1_) on the value of *n*, which is the indication of *L*^QW^ (Fig. 2f, g). In the case of (BA)_2_(MA)_*n*−1_Pb_*n*_I_3*n*+1_ series, the sharp optical absorption edges increase in energy with decreasing *n* value from 1.50 eV (*n* equals ∞) to 2.43 eV (*n* equals 1) in the same manner of the dimension reduction^30,32,33^. When the cation is changed to phenylethylammonium (PEA), the same dimensionality-bandgap relationship is observed^34^. With the decrease of *n* values from ∞ to 1, the energy absorption edge is shifting to higher energy levels. Quan et al. found that when *n* values are decreased, the conduction band minimum (CBM) becomes shallower, while the valence band maximum (VBM) shows a negligible variation. Yao et al.^35^, prepared ‘quasi 2D’ perovskite compounds (PEI)_2_(MA)_*n*−1_Pb_*n*_I_3*n*+1_ by incorporating polyethyleneimine (PEI) cations within the layered structure. In the range of *n* equals 3, 5, or 7, the bandgap is calculated as 1.79, 1.69, and 1.62 eV, respectively. It is worth noting that in this work when *n* equals 3, the resulted *E*_g_ is slightly smaller than that of (BA)_2_(MA)_2_Pb_3_I_10_ due to stronger interactions between the organic−inorganic structures when multi ammonium cation is used^36,37^. These endeavors, taken together, indicate the dimensionality, and resultant optical properties can be controlled in the MDP family (Table 1).

## Interplay between dimensionality, PCE, and stability

Research interests in the adoption of MDPs as a light absorber in PSCs are springing up in recent year. Frequently, two kinds of MDPs are used for the PV application: Class I MDP and Class II MDP (Fig. 1). Class I MDP also called ‘Ruddlesden–Popper hybrid perovskite’ have layered crystal structure and the general formula of ((A)_2_(CH_3_NH_3_)_*n*−1_M_*n*_X_3*n*+1_) (*n* ≤ 60)^12,13^ (Fig. 1). In Class I MDP, the bulky organic cations (A) fragment the compact 3D perovskite structure into isolated inorganic layers of corner-sharing [MX_6_]^4−^ octahedrons with certain layer number (*n*)^20^. The existence of a multiphase system can be unambiguously characterized by X-ray diffraction (XRD) and/or UV absorption. This configuration has been found to increase the formation energy and guard perovskite against attacking from the moisture^34^. As has been shown, the diversified choice of ‘*A*’ and ‘*n*’ endows MD perovskites flexibly tunable and controllable optoelectronic properties.

Smith et al. reported the first demonstration of Class I MDP in PSCs in 2014^11^. They presented a single crystal structure of (PEA)_2_(MA)_2_Pb_3_I_10_ (PEA^+^ denotes phenylethylammonium cation), with dark red color. This is a typical Class I MDP with a clear *n* value of 3 indicating the number of inorganic layers. However, they found that the spin-coated thin film is composed of a mixture of polymorphs with a different number of layers (*n* equals 1, 2, 3, 4, and 5) due to the rapid reorganization process. The MDP based devices showed overall PCE of 4.73%. Despite the low PCE, this MDP displayed excellent moisture resistance even after aging for 46 days under 52% RH, while the MAPbI_3_ based film changed from black to yellow in the meantime (Table 1).

The second Class I MDP was synthesized by Cao et al.^12^, with a general formula of (BA)_2_(MA)_*n*−1_PbI_3*n*+1_ (BA^+^ denotes *n*-butyl-ammonium cation). The single crystal data illustrated vivid, layered structures. To avoid the formation of multiple orders of perovskites in the film for devices, they adopted a low-temperature deposition process. Again, unsatisfactory PCEs of 4.02% were obtained from the Class I MDP with *n* equaling 3 ((BA)_2_(MA)_2_Pb_3_I_10_). Satisfactory stability over two months was observed. In the end, the authors concluded that solution cast Class I MDPs have another problem that the (00l) and (0l0) planes prone to grow parallel to the plane of the substrate, leading to deficient charge transport in the (111) direction (out-of-plane) due to the intercalated insulative organic cation layers. Stoumpos further pushed the limit of *n* in BA/MA mixed Class I MDP to 5 and used the BA_2_MA_4_Pb_5_I_16_ as an active layer in PSC devices^38^. The phase purity was verified by the XRD patterns. The obtained pure compound with *n* equaling 5 was measured to have a direct bandgap (*E*_g_) of 1.83 eV. The planar junction solar cells with the as made thin films demonstrated an improved PCE of 8.71%. These studies with phase pure Ruddlesden–Popper hybrid perovskite^12,13,38^ provided benchmarks facilitating the determination of the composition in a casted film.

So far, the structures of Ruddlesden–Popper type perovskites with *n* from 2 to 5 have been disclosed by X-ray crystallographic technique. With the increase of *n* values (*n* → ∞), the minority bulky cations will be treated as impurities and expelled from the crystal lattice to stay on the surface of the crystal or in the boundary between the crystals. In this case, we call it Class II MDP, which has the ambiguous solid structure without a clearly deduced *n* value (without or with minimum new phase and/or no noticeable change in physiochemical properties)^39^ but maximumly balanced stability versus optoelectronic performance^40^. For example, a thin (BA)_2_PbI_4_ layer formed on the surface and at the grain boundaries of 3D perovskite can enhance the thermal stability of the devices and increase the PCE to 19.56%^41^. In a similar report, a 3D–2D-graded perovskite film resulted in an overall PCE of 19.89%^42^. Very recently, Lee et al.^43^ incorporation of PEA_2_PbI_4_ into the precursor solution of FAPbI_3_. The 2D perovskite spontaneously formed at the grain boundaries of FAPbI_3_ and gave a stabilized PCE of 20.64% (certified stabilized PCE of 19.77%).

Guidelines have been established to select suitable bulky cations for the formation of Class I MDP. Firstly, the bulky organic molecule should contain specific numbers of protonated primary or secondary amines that can efficiently interact with the extended inorganic anionic frameworks through forming hydrogen bonds, yielding 2D LDP with the general formulas of (RNH_3_)_2_MX_4_ or (NH_3_RNH_3_)MX_4_^44,45^. Another structural requirement to form layered perovskites is about the size and shape of the organic parts. Although the bulky molecules step out of the cavity within the 3D AMX_3_ perovskite structure, these molecules should still be able to accommodate themselves within the ‘footprint’ offered by the 2D inorganic framework^46,47^. A recent study by Kamminga et al.^48^ indicated that extending the length of the alkyls in bulky cations can turn on the 1D quantum confinement effect in a 2D quantum confined lead iodide hybrids. The quick crystallization process of MDP normally led to the variation of degrees of quantum confinement, which will create a fluctuating energy landscape and severely limit hole diffusion lengths. Efforts have been put to control over this QW width distribution, and Proppe et al.^49^ showed that higher performance could be achieved by reducing the content of lower *n* QWs. So far, bulky cations like PEA^11^ and BA^12^ are the most popularly adopted candidates in making ‘quasi-2D’ structures when *n* is greater than 1 as the Class I MDP. The criteria for the formation of 1D quantum confined (chained) perovskite frameworks are more complicated and therefore undefined. Medium ammonium cations like EA and PA as well as many Lewis bases type polar molecules (DMSO, DMF, Urea) can slice the lattice of PbX_2_ to form [PbI_*m*_^−^]_∞_ Chains^50^. They are typically used in combination with MA and FA based 3D perovskite to form the Class II MDP^40,51,52^. The formation mechanisms of Class II MDP are more complicated and can be classified into three folds: 2D/3D top capping mode^53^, 2D/3D interspersing mode^54,55^, and interface passivation mode^40,56,57^. Class II MDPs formed from any of these mechanisms contained non-stoichiometric cation ratios, and hence the ‘*n*’ value is undefined and treated as approaching infinite. More examples can be found in Table 1. See Box 1 for the different processing methods toward (a) Class I and (b) Class II MDPs.

### Box 1 Processing methods toward (a) Class I and (b) Class II MDPs

**Processing methods of the Class I and Class II MDPs**

Class I MDP is usually made following the traditional one-step deposition methods, disparate synthesis protocols have been developed to make Class II MDP films, which are typically formed by one-step deposition of non-stoichiometric precursor solutions^40,58^, solution cation exchange^42,51,57,59^, and sequential deposition^56^. There is no defined *n* value and/or evident second phase in the formed solid film. Therefore, we treat in this case the ‘*n*’ value as approaching infinity (quasi-3D).


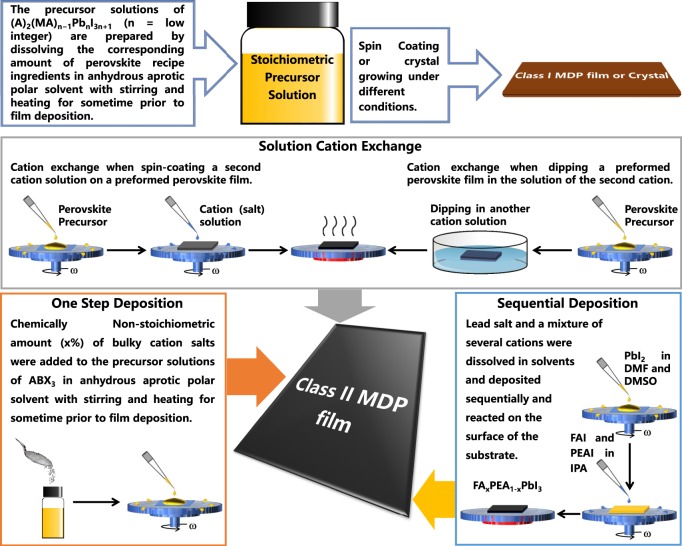


### Enhanced PCE and stability by vertically aligned Class I MDP

**Fig. 3:**
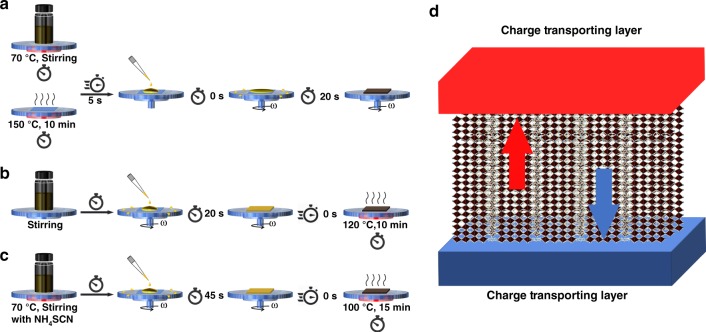
Vertically grown Class I MDPs via different methods. **a** Immediate deposition on the hot substrate; **b** deposited via ‘pre-crystallization annealing’; **c** deposited with NH_4_SCN-modified precursor solution. **d** The resulted MDP films provide direct pathways for charge carriers

To solve the above-mentioned problem of mismatched horizontal orientation of Ruddlesden–Popper type perovskites^60^, Tsai et al.^61^ used so-called ‘hot-casting’ technique to fabricate Class I MDP thin films with near-single-crystalline quality. (Fig. 3a) More importantly, the (00l) and (0l0) planes of the inorganic perovskite component turned out to show prominent out-of-plane alignment preference relative to the substrate electrode and as such facilitating efficient charge transport. (Fig. 3d) The MDP film with a claimed composition of (BA)_2_(MA)_3_Pb_4_I_13_ showed PCE of 12.52% without hysteresis. The devices exhibited significantly enhanced stability compared to their 3D counterparts under the same level of light, humidity, and heat exposure. Unpackaged devices retained more than 60% of their initial efficiency after 2250 h under continuous, standard (AM1.5G) illumination. No degradation was observed when the devices are encapsulated. At the same time, a device based on (BA)_2_(MA)_2_Pb_3_I_10_ (*n* equals 3) showed comparable PCE of 11.44%. Similarly, Zhang et al.^62^, demonstrated Cs^+^-doped (BA)_2_(MA)_3_Pb_4_I_13_-based PSC giving a PCE of 13.7%, the highest among the reported Class I MDP devices. The device doped with 5% Cs^+^ degraded merely 10% after exposure to 30% relative humidity (RH) for 1400 hours, indicating notably enhanced stability under heating and high moisture conditions. The same method was later adopted by several other groups^63,64^. The mechanism and the control of the vertical growth of the Class I MDP is not fully understood until recently when Chen et al.^65^ proposed that the nucleation and growth of vertically orientated 2D perovskite may arise from the liquid-air interface and the ‘pre-crystallization annealing’ (Fig. 3b).

Besides the hot-casting method, Chen and colleagues used NH_4_SCN additive with the optimized ratio to regulate the orientated growth of MDP films with either BA or PEA as the bulky cations. (Fig. 3c) The first try with (BA)_2_(MA)_2_Pb_3_I_10_ and (BA)_2_(MA)_3_Pb_4_I_13_ as absorbers in *p*-*i*-*n*-type devices gave average PCE of 6.82% and 8.79%, respectively, with excellent stability when 1 equivalent NH_4_SCN was added^66^. In a following report, the same group added 2 equivalents of NH_4_SCN into (PEA)_2_(MA)_4_Pb_5_I_16_ and presented the best PCE of 11.01%^67^. Moreover, it is worth mentioning again that the perovskite films reported in the above four pieces of work should be mixed phases with different *n* values as indicated by the extended absorption edges and the position/broadening of photoluminescence spectra. Therefore, it is not scientifically correct to claim as the original work did that the films in the devices were made of MD perovskite with a specific *n* value. All the formulas mentioned above were only used as an indication of the ingredients in the initial recipe.

### The bottleneck in the PCE enhancement of Class I MDP

**Fig. 4:**
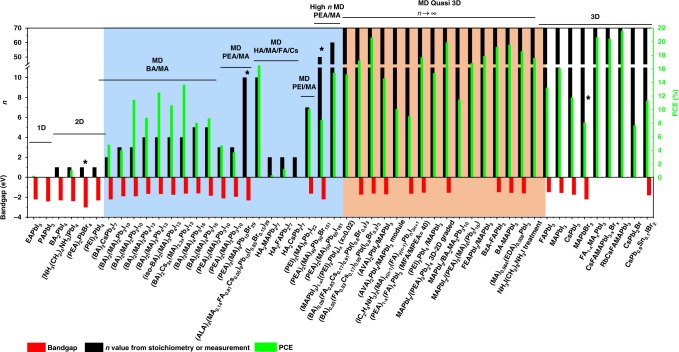
Structure characteristics and device performances of most recent research on MDP and the related PSCs. Data composed based on Table 1 (blue background: Class I MDP; orange background: Class II MDP, asterisk: bromide perovskite)

**Fig. 5:**
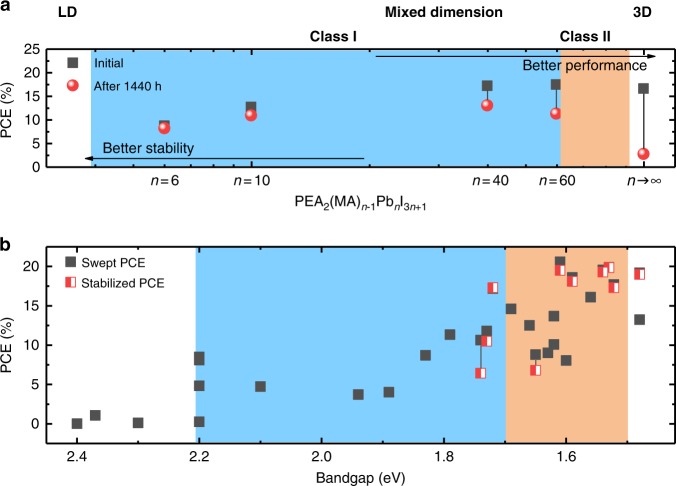
Performance versus *n* values and bandgaps in MDP. **a** Device performance as a function of *n* value. (Reproduced with permission from ref. ^34^) **b** Device performance as a function of bandgaps. (Plotted with data in Table 1, blue background: Class I MDP; orange background: Class II MDP)

While synchronously increased stability and PCE were observed in the Class I MDP based devices, there is an apparent disparity between their PCE and that of 3D perovskites. The reported Class I MDP with well-defined solid structure and/or clearly new phases ended at *n* = 60^34,68^ (Fig. 4). On the left side of Fig. 4, examples of 1D and 2D perovskite are shown. The black bars denote the number of PbI_6_^2−^ layers (*n*). From 1D perovskite to 2D perovskite, the layer number (*n*) increased from 0 to 1. The blue region of Fig. 4 illustrates some properties of Class I MDP and the related devices. In this region, the ‘*n*’ value increase from left to right until 60. The bandgap (*E*_g_) (red bars) is decreasing with the increasing of *n*. With the same *n* value but Br^−^ as the anion, the *E*_g_ is larger than that when the I^−^ is the anion. More importantly, the PCE (green bars) is increasing with the increase of n value, and the BA cation based perovskite gave higher PCEs than PEA based perovskite does. Quan et al. also measured and plotted device performance against the layer number (*n* value), which showed that the performance was increasing with the increase of *n* value, while the stability was decreased in the meantime (Fig. 5a). For example, the devices with 3D perovskite degraded over eight weeks from a PCE of 16.6% to <3% even stored in N_2_, while the performance of the device with *n* equaling 40 with a similar initial PCE dropped to 13.1% after 60 days under the same conditions. Therefore, in the region of Class I MDP, PCEs usually are compromised for the stability with the highest reported PCE of 15.4%, contrasting to the state-of-the-art PCE from 3D perovskite of 23.3%^2,69^. Breakthrough has to be made for such kind of MDPs to improve the PCE and stability further. The correlation between PCE and bandgap energy is plotted in Fig. 5b. The inferior PCE with Class I MDPs is partially due to their wide bandgaps as a result of limited layer number (Fig. 5b).

### The counterpoise between PCE and stability in Class II MDP

**Fig. 6:**
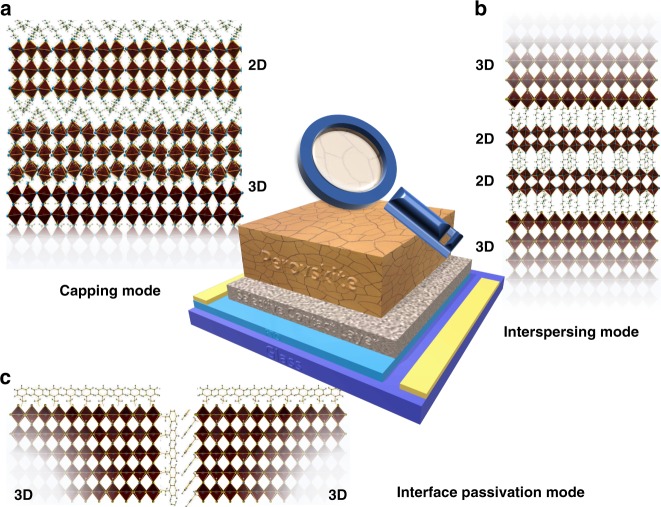
Schematic representation of the two proposed mechanisms to form Class II MDP. **a**, **b** 2D/3D hybrid mode^53–55^; **c** Interface passivation mode^40,56,57^

Lessons learned from the study of Class I MDP pushed the researchers to think about more effective ways of utilizing LDP in the formation of MDPs, namely minority 2D/3D hybrid system and interface passivation. Two synthesis methods can lead to the minority 2D/3D hybrid system: post-ammonium-treatment to form top capping mode and in situ growth to form interspersing mode (Fig. 6a, b). In the case of post-ammonium-treatment, Hu et al.^54^, tried to convert the top layer of the 3D MAPbI_3_ perovskite into an LDP by spin-coating isopropyl alcohol (IPA) solution containing mixed methyl ammonium iodide (MAI) with phenylethylammonium iodide (PEAI) or *n*-butyl-ammonium iodide (BAI) onto the MAPbI_3_ film to form 3D/LDP heterojunction systems (Fig. 6a). Grazing-incidence XRD confirmed the formation of such kind of structure. However, there is no clear border between the two phases, and there is no information about the thickness of each layer. The devices based on a MAPbI_3_/PEAMAPI heterojunction reach PCE of up to 16.84%. They also observed the enhancement in device stability toward exposure to moisture. Bai et al.^42^ treated the 3D perovskite film with a saturated toluene solution of phenylethylammonium iodide (PEAI) instead of pure toluene in the anti-solvent treatment step prior to annealing of the films. Remarkably, they made atomically sharp interface at the border of 3D and 2D perovskite as proved by Tof-SIMS measurement, which was described as the formation of a 2D capping layer together with 3D perovskite or namely 3D–2D graded interface. Like the previous Class II MDP, with this innovation, they realized an excitingly high PCE of 19.89% in a *p*-*i*-*n* device and much-increased moisture/thermal stability as well as suppressed cross-layer ion migration.

As we have introduced in the section about Class I MDP, the *n*-butyl-ammonium (BA^+^) was superior to the PEA^+^ counterparts. However, few examples were shown in the Class II MDP until recently, Wang et al.^53^ tested a range of in situ grown BA_*x*_(FA_0.83_Cs_0.17_)_1−*x*_Pb(I_0.6_Br_0.4_)_3_ with *x* varying from 0 to 1 from the mixed precursor solutions. The authors observed additional reflections in the XRD patterns of MDP perovskites with the increase of the BA concentration. They believe the reflections came from (BA)_2_(FA_0.83_Cs_0.17_)_*n*−1_Pb(I_0.6_Br_0.4_)_3*n*+1_ phases of which the *n* values can not be precisely assigned. (Maybe a mixture of different *n* values) (Fig. 6b) In the end, they achieved a champion stabilized PCEs of 19.5% and 17.3% based on MD perovskites with a bandgap of 1.61 and 1.72 eV, respectively. They claimed the observation of 2D perovskite platelets interspersing between highly orientated 3D perovskite grains perpendicularly to the plane of the film, which suppresses non-radiative charge recombination. This film is realized by a post burn-in procedure (175 °C) similar to the method in Fig. 3c and can be seen as a composite of 2D and 3D perovskite. In the end, they reported enhanced stability under simulated sunlight. Devices sustain 80% of their ‘post-burn-in’ efficiency after 1000 h in air, and close to 4000 h when encapsulated.

Another proposed mechanism is called interface passivation, which means the introduced bulky cations did not induce the formation of a new phase but only anchored on the surface or intercalated between the grain boundary to form a passivation layer (Fig. 6c). The first typical example is given by Zhao et al.^62^, who introduced selected diammonium iodide by post-treating 3D (MAPbI_3_). The difference in this study from previously discussed reports is that they used rigid NH_3_I(CH_2_)_8_NH_3_I (C8) instead of short BA or flexible NH_3_I(CH_2_)_2_O(CH_2_)_2_NH_3_I (EDBE). Instead of inducing 3D to 2D perovskite phase transformation, the C8 diammonium iodide can successfully passivate perovskite surface (I vacancy) and grain boundaries (GBs) due to the elevated activation energy arising from its unique anti-gauche isomerization. The passivated device showed a much-improved PCE of 17.60% compared to the control device showing a PCE of 14.64%. Later, Lu et al.^62^ used comparatively low concentrations of diammonium cations to prevent the formation of pure 2D structures. In this case, the addition of an insignificant amount of short ethane-1,2-diammonium (EDA^+^) (e.g., 0.8 mol%) into the precursor solution, which did not induce the formation of a new phase but improved the PCE under one sun irradiation from 16.7% ± 0.6% to 17.9% ± 0.4% (aperture of 0.16 cm^2^). The authors demonstrated certified PCE of 15.2% ± 0.5% with the large active area (aperture 1.04 cm^2^).

Moreover, the in operando stability test of EDA^2+^/MA^+^-based solar cells showed 75% retention of the initial PCE after 72 h at 50% RH and 50 °C under one sun illumination, while the MAPbI_3_-based devices degrade by 90% after 15 h. Gao and colleagues^40^ used a novel fluorinated aliphatic cation (trifluoroethyl amine iodide) to modify MAPbI_3_ perovskite and enhance the environmental stability without compromising the performance of the devices. After introducing 3 mol% FEAI additives into the perovskite precursor solution, the average PCE of the mesoporous PSC significantly increased from of 15.6 to 18.0%. Due to the hydrophobic nature of the CF_3_-terminal group over the surface of the 3D perovskite, moisture resistance is enhanced without forming wide *E*_g_ 2D perovskite, which represents a new paradigm of stabilizing PSCs.

Phenylethylammonium (PEA) is the first bulky cation used in Class I MDPs^11^ and the highest PCE achieved in Class I MDP (*n* equals 60) was around 16% from one-step deposition method^34^. Recently, Li et al.^56^, deposited mixed solution of FAI/PEAI (M_FAI_/M_PEAI_ equals 40) atop preformed PbI_2_ film to convert it into perovskites. Instead of forming a new phase, the PEA^+^ self-assembled on both the surface and grain boundaries of 3D FAPbI_3_ via induced *π*–*π* interactions, which can increase the phase transition energy and reinforce phase stability. As a result, a high PCE of 17.7% with superior phase and ambient stability was successfully demonstrated. Wang et al.^51^, further entrusted various phenylalkylamines the mission of moisture resistance. They introduced directly 3 vol% of basic amines (not ammonium salt) in chlorobenzene onto the preformed 3D FAPbI_3_ by spin-coating. No new phase was detected, and the presence of the passivation layer and its chemical bonding to the perovskite can only be confirmed by the Fourier transform infrared spectroscopy (FTIR) and 1H nuclear magnetic resonance (NMR) measurements. With this method, they successfully fabricated PSCs using benzylamine treatment with PCEs above 19% and kept stable in moisture air for more than four months. Coincidentally, Lin et al.^41^ compared the effect of treatments by either *n*-BA or *n*-BAI and they got PCEs around 19% from both cases. At the far right of Fig. 4 and bottom of Table 1, parameters of typical 3D perovskites including stability test information were listed for comparison. Among them, the mixed-cations 3D perovskite gave excellent PCE above 22%. Apparently, the Class II MDP combined the merits from both 3D perovskites (high PCE) and Class I MDP (high stability), with the highest PCE above 19% with the equally competitive stability (Table 1, Fig. 4).

## Stability enhancement with MDP

### Intrinsic and extrinsic factors

**Fig. 7:**
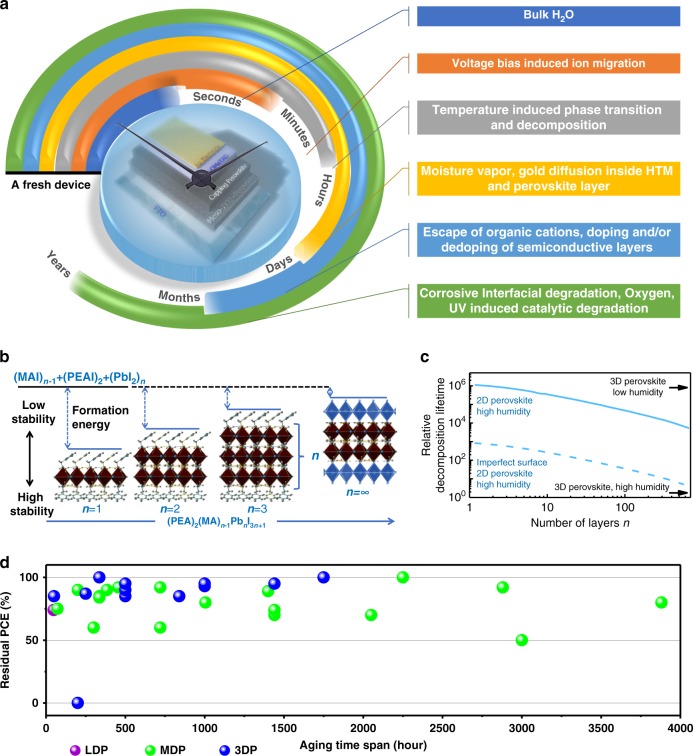
Degradation analysis of a PSC device and the influence of MDP strategy. **a** Examples of intrinsic and extrinsic degradation mechanisms that lead to device failure at different timescales on a fresh hybrid perovskite device. **b** Energetics of perovskite formation and stability. Unit cell structure of (PEA)_2_(MA)_*n*−1_Pb_*n*_I_3*n*+1_ perovskites with different n values, showing the evolution of dimensionality from 2D (*n* = 1) to 3D (*n* = ∞) (Reproduced with permission from ref. ^34^); **c** DFT simulation of the decomposition lifetime with different *n* values in different atmospheres (Reproduced with permission from ref. ^34^; **d** Summary of stability test results. Data composed based on Table 1. (red dot: 1D and 2D perovskite; green dot: MDP; blue dot: 3D perovskite; each spot represents one residual PCE after certain test time span under one stability test condition)

The degradation pathways of a PSC device can be complicated and related to many factors from all the components. Generally, they are unstable when exposed to humidity, high temperatures, and light^70,71^. The factors inducing degradation in PSCs can be classified as intrinsic and extrinsic, which take effect under different timescale as illustrated in Fig. 7. To be specific, the extrinsic factors that can lead to degradation include H_2_O (moisture), voltage bias stimulus, temperature, photon stimulus, oxygen, etc. that come from the ambient environment. While the intrinsic instable factors include the hygroscopicity and volatile organic cations, ion conductive nature of perovskite material, solid–solid diffusion. To destroy a PSC device, the extrinsic and intrinsic factors sometimes take effect alone and sometimes there are synergistic effects from both the extrinsic and intrinsic factors, for example, the UV-induced catalytic degradation. Most extrinsic factors can destroy the device in a relatively short timescale, while intrinsic factors usually are working during a longer timescale. Firstly, in a few seconds, the device can be failed upon exposure to bulk water^72^. Then under working conditions, the voltage bias can lead to macroscopic migration of a large amount of I^−^ ion in minutes^73^. Moreover, the temperature has a significant impact on the structural stability of the perovskite from two different aspects. For one thing, a variation of temperature transforms the symmetry of crystal from orthorhombic to tetragonal and finally to cubic^74^; for another, further, increase the temperature can destroy the perovskite film and leave PbI_2_ behind in hour-timescale^75^. For a similar reason, under thermal stress, the element of the metal electrode (e.g., Au, Ag, Al, etc.) can migrate following the Fick’s Law^76^ across the hole-transporting layer (HTL) and into the perovskite layer after days, which will severely affect the in operando device performance^77,78^. The water vapor absorbed from the ambient can hydrolyze the perovskite and form activated cations such as CH_3_NH_2_, which escape slowly from the surface of the perovskite film. Meanwhile, the exposure of the film to oxygen and light over months can further accelerate the decomposition of the cation salt and hydroiodide and generate more and more free iodine, which can dope the active layers^79^. With encapsulation, light exposure induced decomposition of perovskite at the interface between metal oxide based photoanodes (e.g., TiO_2_, SnO_2_ etc) and perovskite can be slowed down, which will however still happen over the years due to their photocatalytic effects^80^. All the above degradation mechanisms inseparably interconnected, and the short timescale mechanisms usually promote long timescale mechanisms^81,82^.

### Degradation mechanism

The motivation behind the rising of MDPs is the potential to increase the operating stability but not necessarily compromise the PCE. Piles of works have proved that the MDP strategy did improve the stability of the PSCs. To identify the mechanism behind the MDP materials, Quan *et al*., studied the stability of dimensionally tuned Class I MDPs using density functional theory (DFT) simulations, and calculated formation energies (*E*)^34^. (Eq. 1) Calculations show that the *E* value and decomposition lifetimes are decreasing with the increasing of n value. While conventional 3D perovskites have the lowest formation energy, therefore they tend to reversely decompose into PbI_2_ and MAI constituents, as have been evidenced by experiments^83^. This process starts from the surface of the material, where molecules have sufficient freedom to rearrange.

1 $$E = \frac{{E_{({\mathrm{PbI}}_2)_n({\mathrm{MAI}})_{n - 1}({\mathrm{PEAI}})_2} - nE_{{\mathrm{PbI}}_2} - \left( {n - 1} \right)E_{{\mathrm{MAI}}} - 2E_{{\mathrm{PEAI}}}}}{n}$$

Since the predicated formation enthalpy per molecule required to recrystallize the MAI layer is about 0.55 eV but −0.6 eV for PbI_2_, MAI capping is serving as a protection layer, while the PbI_2_ surface termination is inherently unstable^84^. On the other hand, the calculated energies needed for desorption of MAI and PEAI from perovskite into gas phase are 2.15 and 2.51 eV, respectively. MAI is, therefore, easier to escape from the solid surface than PEAI to leave unstable PbI_2_ surface behind. According to the differences (0.36 eV) between the energy required to remove PEAI from the perovskite and that for MAI, they conclude that the adoption of MDP (either Class I or Class II) over 3D Perovskite can reduce the desorption rate by six orders of magnitude, and slowdown film decomposition by 1000-fold. Figure 7c shows that an increased performance can achieve when increasing the *n* value; however, in the meantime, stability is decreasing. There is a region where MDP can find a balance between PCE and lifetime.

### Lifetime tests

An immediate example was shown recently by Grancini that one-year stable perovskite devices can be realized by engineering an ultra-stable Class II MDP (AVA)_2_PbI_4_/CH_3_NH_3_PbI_3_ (AVA: 5-ammoniumvaleric acid) perovskite junction^58^. A gradually organized multi-dimensional interface is formed and showed PCE up to 12.9% in a carbon-based architecture, and 14.6% in standard mesoporous solar cells. More importantly, the 10 × 10 cm^2^ solar modules by a fully printable industrial-scale process, delivering 11.2% efficiency with zero loss during the measurement period (more than 10,000 h). This is so far the most extended lifetime test on a PSC solar module (Table 1). In Fig. 7d, a correlation between the PCE drop versus aging time span is plotted based on data in Table 1 about different types of perovskites. Without considering the testing conditions like humidity and temperature, the data shows that although 3D perovskites were reported with unexpectedly good stability under test, MDP perovskites were tested with the twice longer time span.

Although the ever-extending span of lifetime test is exciting, one must be soberly aware that most of the stability measurements were not carried out under the same standard conditions, e.g., unified relative humidity, temperature, light intensity, atmosphere and the same fitting function of PCE retention. Therefore, it is hard to compare the results of two lifetime tests from two different reports from different or even the same lab(s). Moreover, most of the reported time-dependent change of PV parameters were based on normalized (to 1) data of arbitrary cells, which can hardly be related to the stability of the champion devices. Given the facts above, it is necessary to consolidate the testing conditions for the evaluation of the stability of a perovskite solar cell. It is well known that there exist the standard damp heat conditions (85^o^C and 85% relative humidity (RH) for 1000 h) as related to International Electrotechnical Commission (IEC) test 60904^85^ and 61646^86^, so far, however, most of the lab-made devices were obviously tested under various customized conditions^87^. (Table 1) It is recommended that all the devices that are promising be tested under 100 mW cm^−2^ 1.5 AM illumination with standard damp heat conditions. Then we can screen out and identify the most promising perovskite recipes and device engineering methods.

## Conclusions and perspectives

MDPs have been accepted as potential candidates to circumvent the dilemma of high performance but low ambient instability with conventional 3D perovskites. Comparing to pure 3D and LD perovskites, multi-dimensional perovskites (MDP) maintain balanced performances, bandgaps, charge transporting properties, and ambient stability, which are critical for photovoltaic applications. After the meticulous analysis of the reported data, two important points should be considered during the design of the new MDP. Firstly, techniques like hot-casting or so-called ‘pre-crystallization annealing’ should be adopted to induce vertical growth of perovskite crystallites since the intrinsic 2D perovskites tend to grow parallel to the plane of the substrate and the charge transfer efficiency in the normal direction (to the charge selective contact layers) is very low. This is very important in the case of Class I MDP and so far, PCEs of above 15% have been realized with notable long-term stability using this method^62^. However, one should keep in mind that hot-spin-casting is not compatible with large-scale processing. It may be possible to integrate the ‘pre-crystallization annealing’ into techniques like roll-to-roll printing or doctor-blading. In the meantime, the mechanism of additive induced oriented growth of perovskite should be further studied. If the same type of morphology, the namely vertical growth of MD perovskite crystallites, can be realized, the mass production of perovskite solar module will benefit from such stable perovskite material.

The second point is about the interfacial and grain boundary engineering in Class II MDP. Instead of adding the chemical stoichiometric amount of bulky cations, Class II MDP includes the MDPs with partially substituted MA^+^ or FA^+^ on the surface of grain by bulky, hydrophobic organic cations. This strategy has been affirmed as one of the most effective ways to improve the stability of perovskites without sacrificing device performances. So far the Class I MDPs with Ruddlesden–Popper perovskite structure have provided devices with improved stability and moderate PCEs, whereas interfacial modifications and passivation in Class II MDPs further push the device PCE up to 20% and on par with 3D perovskite^53^. Mechanisms behind the remarkably enhanced long-term stability have been simulated and proved by experiments. In this review, based on the analysis of the most updated research results, we anticipate that even stronger motivation and more efforts will be invested in the research of new MDP with new hydrophobic cations for both higher PCEs and stability. The stability test conditions should be standardized to the industry conditions, and better stability performance should be realized with MDPs (<5% loss in PCE after tested at 85^o^C and 85% RH for 1000 h). Sharing the merits of the low-cost manufacturing property of 3D perovskites, such as roll-to-roll slot die-coating, screen-printing, or ink-jet printing technologies, large area MDP modules with improved ambient stability will be the real breakthrough and challenger to the state-of-the-art Si-based PVs.
